# Association of complement factor H Y402H polymorphism with phenotype of neovascular age related macular degeneration in Israel

**Published:** 2008-10-08

**Authors:** Itay Chowers, Yoram Cohen, Nitza Goldenberg-Cohen, Joaquin Vicuna-Kojchen, Alejandro Lichtinger, Orly Weinstein, Ayala Pollack, Ruth Axer-Siegel, Itzhak Hemo, Edward Averbukh, Eyal Banin, Tal Meir, Michal Lederman

**Affiliations:** 1 Department of Ophthalmology, Hadassah–Hebrew University Medical Center, and the Hebrew University School of Medicine, Jerusalem, Israel; 2 Cancer Research Center, Sheba Medical Center, Tel Aviv University, Tel Aviv, Israel; 3 Department of Ophthalmology, Rabin Medical Center, Petah Tiqva, Israel; 4 Department of Ophthalmology, Soroka University Medical Center, Beer Sheva, Israel; 5 Department of Ophthalmology, Kaplan Medical Center, Rehovot, Israel

## Abstract

**Purpose:**

The Tyr402His variant of complement factor H (CFH) is associated with age-related macular degeneration (AMD) in several populations. Our aim was to evaluate if this single nucleotide polymorphism (SNP) is associated with AMD in the Israeli population and see if it underlies heterogeneity in clinical manifestation and responses to photodynamic therapy (PDT), which characterize neovascular AMD (NVAMD).

**Methods:**

Genotyping for the Tyr402His variant was performed in 240 NVAMD patients (78.1±7 age range) and 118 controls (70.8±8.2 age range). Genotyping was correlated with clinical characteristics and treatment parameters in sequential 131 NVAMD patients who underwent PDT.

**Results:**

TheTyr402His coding allele was associated with NVAMD in the Israeli population: odds ratio (OR)=1.9; 95% confidence interval (CI)=1.3–2.6; p=0.0002. Homozygosity for this variant was associated with an OR of 3.4 (95% CI: 1.7–6.8) for having AMD. There was no association among this SNP and age of onset of NVAMD, gender, neovascular lesion size, initial or final visual acuity, and number of PDT sessions required.

**Conclusions:**

In accordance with findings from the majority of previous study populations, the Tyr402His variant of CFH is associated with NVAMD in Israel. However, heterogeneity in clinical manifestations of NVAMD and in its response to PDT is not underlined by this CFH variant and may be accounted for by other genetic and environmental factors.

## Introduction

Genetic factors play a strong role in the pathogenesis of age related macular degeneration (AMD). While variations in the sequence of several genes have been associated with AMD in recent years [1], single nucleotide polymorphism (SNP) in the gene for complement factor H (CFH) appears to be one of the most consistent and important genetic risk factors for AMD [2-8].

The Tyr402His variant of CFH (encoded by the C allele of rs1061170 SNP) has been found to be associated with AMD in several populations worldwide [3,5,6,9,10], but its association with AMD in the Israeli population is unknown. Subsequently, additional variants in both coding and noncoding regions of the CFH gene, which are associated with either increased or decreased, risk for developing AMD, have been identified [11,12]. In view of CFH known function in maintaining homeostasis of the complement system combined with evidence for involvement of inflammation in the pathogenesis of AMD, it is likely that altered function of CFH variants affecting inflammatory response may account for its association with the disease [13].

While genetic factors such as SNPs in CFH may increase the likelihood of an individual to develop AMD, it is unclear if these genetic factors also underlie variations in the clinical manifestations of neovascular AMD (NVAMD) such as variable age of onset, neovascular lesion size, visual acuity, and response to therapy. Recent studies have suggested that homozygosity for the Tyr402His variant of CFH may be associated with classic or predominantly classic choroidal neovascularization (CNV) lesion type according to fluorescein angiography [14-16], and with response to bevacizumab therapy [17]. Conflicting evidence were reported with respect to the association of the same variant and response to photodynamic therapy (PDT) [15,18].

To further assess this issue we first evaluated the association among NVAMD and the Tyr402His CFH variant in the Israeli population. We then studied the correlation among this variant, phenotype, and outcome following PDT.

## Methods

The study included 240 NVAMD patients recruited from four retina clinics in Israel and 118 unaffected controls who were evaluated for routine eye examination, or for pathologies other than AMD, in the Department of Ophthalmology of the Hadassah–Hebrew University Medical Center in Jerusalem, Israel. Institutional Ethics Committee approval was obtained for the study, and each patient signed an informed consent form. AMD was diagnosed and graded according to the AREDS trial classification [19]. Inclusion criteria for the control group included age over 60 years, clear media which enabled ophthalmoscopy, and absence of intermediate size drusen, multiple small drusen, or retinal pigment epithelial abnormalities.

The female to male ratio was balanced between AMD patients and controls. The mean age in the controls (70.8±8.2) was lower than that of AMD patients (78.1±7.6, p<0.05, *t*-test). The control group included 10 Arabs, 40 Sephardic Jews, and 67 Ashkenazi Jews, while the study group included 7 Arabs, 73 Sephardic Jews, and 154 Ashkenazi Jews (p=0.047, χ^2^ test). The ethnicity of one individual from the control group and six NVAMD patients was unknown. Median follow-up of NVAMD patients having PDT was 16 months (range 1–156 months).

Detailed clinical information was available on a sequential subgroup of 131 NVAMD patients (of the 240 patients enrolled in the study), who were treated with PDT at the Hadassah–Hebrew University Medical Center. These patients were included in the phenotype-genotype analysis. Fluorescein angiograms of these NVAMD patients were reviewed by retina specialists (I.C., E.B., I.H., and E.A.) who were masked with respect to genotyping results. CNV was classified as classic, predominantly classic, minimally classic, or occult based on the guidelines of the Macular Photocoagulation Study Group [20]. Retinal angiomatous proliferation (RAP) lesions were classified as occult lesions. Review of the entire group of patients was also performed in a masked fashion (with respect to previous lesion type classifications and to genotypes) by one of the investigators (I.C.). There was agreement in 83.4% of cases between this investigator and the classification by the treating retina specialist (kappa measurement of agreement=0.68; p<0.0001). Classification according to the retina specialist which review the entire cohort was used for statistical analysis. The standard PDT protocol for NVAMD was applied [21].

Genotyping for the rs1061170 SNP on the gene for CFH was performed using iPLEX^TM^ chemistry on a matrix-assisted laser desorption/ionization time-of–flight (MALDI-TOF) mass spectrometer (Sequenom Inc., San Diego, CA).

Statistical analysis was performed using the SPSS (SPSS, Chicago, IL) and Instat software (GraphPad, San Diego, CA). Logistic regression and χ^2^ tests were applied to assess odds ratios, confidence intervals, and significance.

Based on the number of individuals included in the analysis, this study had 85% power for identification of an association between genotypes and lesion type in the magnitude that was described by Brantley and colleagues [14]. Our study also had 94% power for identification of an association between genotypes and visual acuity following PDT in the magnitude described by Brantley and colleagues [22].

## Results

The rs1061170 CFH SNP was in Hardy–Weinberg equilibrium. Distribution of the genotypes was significantly different between NVAMD patients and controls (p=0.00047, χ^2^ test). Homozygotes for the C allele of rs1061170 had an odds ratio (OR) of 3.4 and a 95% confidence interval (CI) of 1.7–6.8. Heterozygotes had an OR of 2.1 (95% CI 1.3 −3.4) for having NVAMD compared with homozygotes for the wild-type allele, respectively (Table 1). Combined, individuals either homozygous or heterozygous for the C allele had an OR of 2.4 (95% CI 1.5–3.8, p=0.0005) compared with individuals homozygous for the T allele for having AMD. Analysis according to distribution of the C and T alleles showed similar findings (Table 1). We evaluated if the absence of drusen in some of the NVAMD cases could introduce bias and reduce the strength of the associated between rs1061170 and NVAMD in our study.

**Table 1:** The frequency of alleles and genotypes of complement factor H rs1061170 SNP in Israeli NVAMD patients and unaffected controls

**Population **	**Age-related macular degeneration**	**Unaffected**	**p value**	**OR (95% CI)**
**By allele (T/C)**				
Entire population	237/243 (49.4%/50.6%)	152/84 (64.4%/35.6%)	0.0002	1.9 (1.3–2.6)
Ashkenazi Jews	147/161 (47.7%/52.3%)	87/47 (64.9%/35.1%)	0.0009	2 (1.3–3.1)
Sephardic Jews	74/72 (50.6%/49.4%)	51/29 (63.7%/36.3%)	0.07	1.7 (1–3)
**By genotype**				
Entire Population				
TT	55 (22.9%)	49 (41.5%)	0.00047	
TC	127 (52.9%)	54 (45.8%)		2.1(1.3–3.4)
CC	58 (24.2%)	15 (12.7%)		3.4 (1.7–6.8)
**Ashkenazi Jews**				
TT	33 (21.4%)	27 (40.3%)	0.003	
TC	81 (52.6%)	33 (49.3%)		1.9 (1–3.8)
CC	40 (26%)	7 (10.4%)		13.9 (3.1–62.5)
**Sephardic Jews**				
TT	18 (24.7%)	17 (42.5%)	0.19	
TC	38 (52%)	17 (42.5%)		1.8 (0.7–1.9)
CC	17 (23.3%)	6 (15%)		2.8 (0.9–9.5)

Comparison of the frequency (%) of complement H rs1061170 variants between NVAMD patients and controls in the entire Israeli population and in the Ashkenazi and Sephardic subpopulations. Increased prevalence of the C variant was associated with the disease in the Ashkenazi subpopulation. CI- confidence interval, OR- odds ratio.

Of 240 NVAMD patients, 234 had drusen, three did not have drusen, and in three patients the presence of drusen could not be confirmed due to bilateral large exudative lesions. The association between NVAMD patients with drusen and the C allele was similar in magnitude to the association between the entire NVAMD cohort and the same variant (data not shown).

A subgroup analysis was performed to evaluate if the rs1061170 SNP is associated with NVAMD among Ashkenazi and Sephardic Jews. There were too few individuals of Arab origin in our cohort to evaluate for an association between rs1061170 and NVAMD in this ethnic group. The Tyr402His variant was associated with NVAMD among Ashkenazi Jews (p=0.003, χ^2^ test). There was a trend toward such an association among Sephardic Jews (p=0.19, χ^2^). Analysis according to alleles showed similar findings (Table 1).

Following the establishment of an association between rs1061170 and NVAMD in our population, we have correlated genotypes with clinical characteristics of NVAMD and with response to PDT. Included in this analysis were 131 sequential NVAMD patients, who were treated in the Department of Ophthalmology of the Hadassah Medical Center and who were characterized in terms of phenotype and response to PDT. There were no associations found between rs1061170 genotypes and clinical or demographic parameters of NVAMD patients including age, gender, AMD family history, lesion size, initial and final visual acuity, and number of PDT sessions required.

Genotype distribution was significantly associated with lesion type when classified into classic (including pure classic and predominantly classic) or occult (pure occult and minimally classic) lesions (Table 2). Yet, while heterozygotes had a 2.5-fold higher prevalence of occult than classic lesions, the proportion of such lesions was nearly balanced in homozygotes for either the T or C alleles (Table 2). Patients were then classified to those carrying the C allele (either homozygotes or heterozygotes) and those that are homozygotes for the T allele. Lesion type was not associated with this classification (p=0.23). Analysis according to four categorizes (classic, predominantly classic, minimally classic, occult) showed similar findings (data not shown). There was no association between genotypes and response to PDT when assessed separately in classic (including predominantly classic) and occult (including minimally classic) lesions.

**Table 2:** Evaluation for association among clinical parameters in patients with NVAMD and complement factor H rs1061170 variants

**Parameter **	rs1061170 **genotype**			
	**TT**	**CC**	**TC**	**P**
Gender (female/male)	13/12	10/25	37/34	0.057
Lesion type (classic/occult)*	14/11	16/19	20/51	0.027
Family History of AMD (yes/no) ^#^	2/23	6/21	11/47	0.353
Age (mean ± SD, in years)	78.4±9.63	78.5±7.44	78.68±7.89	0.989
Initial VA (mean±SD, logMAR)	1.1±0.82	1.15±0.91	1±0.71	0.659
Lesion size (mean±SD, in µm)	3965.7±1711.4	4074.4±1386.9	3580.1±1019.1	0.207
Number of PDT sessions	2.76±2.47	2.06±1.92	2.06±1.56	0.240
Final VA (mean±SD, logMAR)	1.53±0.98	1.53±0.95	1.35±0.84	0.547

Analysis for potential association among the wild type (T) and risk (C) alleles of the rs1061170 single nucleotide polymorphism in complement factor H and clinical parameters in patients with NVAMD is presented. There was no association between homozygosity for the risk allele and the factors which were evaluated. The asterisk represents that despite the presence of an association among heterozygosity and lesion type, there was no association between lesion type and rs1061170 when comparing lesion type in patients with TC and CC genotype combined with lesion type in patients with TT genotype, or when comparing lesion type between patients with the TT and CC genotypes. The following abbreviations and symbols are used: visual acuity (VA), photodynamic therapy (PDT); the sharp(hash mark) represents family history for age-related macular degeneration could not be reliably assessed for 21 patients.

## Discussion

We found that the Tyr402His variant of CFH is associated with NVAMD in the Israeli population. Association of the Tyr402His variant of CFH with AMD has been described in several populations worldwide [3,6,9,10,23-26], but it appears to be less common in Chinese [27], and is not present in Japanese [28-30]. The composition of the Israeli population, which was included in the present study, is different from previously studied populations. Our study group is composed of several ethnic groups, which may be classified into three main groups: Arabs, Sephardic Jews, and Ashkenazi Jews. Our data suggest that the Tyr402His variant is associated with NVAMD in Ashkenazi Jews. A trend toward such an association was observed among Sephardic Jews, and the distribution of genotypes in this group suggests that the magnitude of the association of the Tyr402His variant is similar in Ashkenazi and Sephardic Jews. There were too few Arab NVAMD patients in this study to determine if the Tyr402His variant is associated with NVAMD in this ethnic group. The relatively low number of Arabs in the study group compared with the control group may stem from the rarity of NVAMD in the Arab population in our referral area [31].

A stronger association between NVAMD and the Tyr402His variant compared with the one we have documented was demonstrated in certain but not in all cohorts [5,23,32,33]. For example, Haines and colleagues [5] described an OR of 2.45 for heterozygotes and an OR of 3.33 for homozygotes of the Tyr402His variant among AMD patients. These respective ORs increased to 3.45 and 5.57 among patients with NVAMD [5]. Yet, a recent meta-analysis of 14 studies revealed an average OR of 2 for the association between the C allele encoding the Tyr402His variant and AMD, a value similar to the one we detected (OR=1.9) [34]. Thus, it is unclear if there are differences strength of association between the Tyr402His variant and AMD among Israeli population and white populations.

Inflammation is thought to play a role in CNV [35-37], and an altered inflammatory response associated with the Tyr402His variant may theoretically modulate CNV as well as affect its response to therapy. In our study, this potential effect of Tyr402His variant on NVAMD had no apparent clinical implications. Diverse clinical characteristics of NVAMD among patients such as age of onset, initial visual acuity, lesion size, and response to PDT were not attributable to the Tyr402His variant.

An increased prevalence of occult CNV among heterozygotes was observed in our study, but the importance of this association is unclear. Homozygotes for the wild-type and risk alleles had a similar prevalence of occult and classic lesions, and there was no association between lesion type and the Tyr402His variant when carriers for the variant (homozygote and heterozygotes combined) were compared with homozygote for the wild-type allele. An association between the Tyr402His variant and classic CNV lesion type was previously reported in three cohorts, while two other studies failed to identify such an association. One study reported that the same variant may be associated with occult lesion type [14-17,38,39]. Combined, these data suggest that the Tyr402His variant does not have a major contribution for determination of lesion type. Additional studies of different populations are required to determine if the Tyr402His variant is indeed associated with lesion type and if such an association is population-specific.

Revealing such pharmacogenetic interactions in AMD may facilitate improved treatment selection for the individual patient and provide important insight into the pathogenesis of the disease. Interactions between the Tyr402His variant and PDT or anti-vascular endothelial growth factor (VEGF) compounds, if they exist, should be reflected in parameters such as the visual outcome following applications of these therapies or number of treatment sessions required. Goverdhan and colleagues [15] reported that the degree of visual loss following PDT was significantly higher in homozygotes for the Tyr402His variant among 27 NVAMD patients who had PDT. By contrast, Brantley and colleagues found that among 69 NVAMD patients, the post-PDT visual acuity was better in homozygotes for the Tyr402His variant compared with homozygotes for the wild-type allele [22]. However, in accordance with our findings in 131 NVAMD patients, Seitsonen and colleagues [40] did not find an association between the Tyr402His variant and response to PDT among 88 NVAMD patients. Thus, should interactions between PDT and the Tyr402His variant exist, they may be population-specific. While the association between PDT and Tyr402His variant is still unclear, Brantley and colleagues [17] reported that among 86 NVAMD patients treated with bevacizumab injections, homozygotes for the Tyr402His variant had worse visual outcome. Thus, pharmacogenetic interactions in NVAMD may exist and may potentially be treatment- and perhaps population-specific.

Elucidating the genetic or environmental factors that underlie heterogeneity in the manifestation and response into treatment of NVAMD will provide important insight to the pathogenesis of the disease and may enable selection of the most appropriate therapy for each patient. Our data did not demonstrate a major contribution of the Tyr402His polymorphism in the course of NVAMD and its response to therapy in the context of other genetic backgrounds and environmental conditions.
